# Formulation of Folate-Modified Raltitrexed-Loaded Nanoparticles for Colorectal Cancer Theranostics

**DOI:** 10.3390/pharmaceutics12020133

**Published:** 2020-02-05

**Authors:** Justin G. Rosch, Allison N. DuRoss, Madeleine R. Landry, Conroy Sun

**Affiliations:** 1 Department of Pharmaceutical Sciences, College of Pharmacy, Oregon State University, Portland, OR 97201, USA; roscju@ohsu.edu (J.G.R.); duross@ohsu.edu (A.N.D.); landrym@ohsu.edu (M.R.L.); 2 Department of Radiation Medicine, School of Medicine, Oregon Health & Science University, Portland, OR 97239, USA

**Keywords:** folic acid, raltitrexed, tangential flow filtration, nanoparticles, NIR imaging

## Abstract

Multifunctional nanoparticles (NPs) that enable the imaging of drug delivery and facilitate cancer cell uptake are potentially powerful tools in tailoring oncologic treatments. Here we report the development of a layer-by-layer (LbL) formulation of folic acid (FA) and folate antimetabolites that have been well-established for enhanced tumor uptake and as potent chemotherapeutics, respectively. To investigate the uptake of LbL coated NPs, we deposited raltitrexed (RTX) or combined RTX-FA on fluorescent polystyrene NPs. The performance of these NP formulations was evaluated with CT26 murine colorectal cancer (CRC) cells in vitro and in vivo to examine both uptake and cytotoxicity against CRC. Fluorescence microscopy and flow cytometry indicated an increased accumulation of the coated NP formulations versus bare NPs. Ex vivo near-infrared (NIR) fluorescence imaging of major organs suggested the majority of NPs accumulated in the liver, which is typical of a majority of NP formulations. Imaging of the CRC tumors alone showed a higher average fluorescence from NPs accumulated in animals treated with the coated NPs, with the majority of RTX NP-treated animals showing the consistently-highest mean tumoral accumulation. Overall, these results contribute to the development of LbL formulations in CRC theranostic applications.

## 1. Introduction

Folic acid (FA) is a metabolite required for DNA synthesis in the proliferation of new cells [1]. This small molecule, comprised of a pterin, p-amino benzoic acid, and glutamic acid [2], has been investigated as a targeting agent for molecular and nanoparticle-based therapies due to the prevalence of folate receptors and carriers present on various cancer cells [3,4,5,6]. By attaching FA to a drug carrier, direct targeting can potentially result in enhanced uptake in tumors [5,7,8], especially those with over-expression of the α-folate receptor [6].

Anti-folate drugs have been a common strategy for targeting various cancer cells. Methotrexate was one of the first of these compounds to see wide clinical use, but suffers from low aqueous solubility. Methotrexate inhibits dihydrofolate reductase, a necessary component in purine synthesis [9]. Therapeutic strategies evolved to target thymidylate synthase, which is also required for cell proliferation. Raltitrexed (RTX) and pemetrexed, among others, were compounds that saw use as a result of drug design and development in this area [9,10,11]. Potency varies, but is generally higher than early anti-folate drugs, with reduced side effects (such as nephrotoxicity), and more specific binding to their targets (α-folate/β-folate receptors and the reduced folate carrier) [6]. 

This class of drugs has been employed in the treatment of numerous cancers, including colorectal [12], lung [13], and pancreatic [14], among others, and usually in combination with other chemotherapy. Of particular note for this work is RTX’s use as adjuvant therapy in the treatment of colorectal cancer (CRC) [15,16,17,18,19,20]. Nanoparticle-based delivery of therapeutics offers numerous benefits over parenteral administration of comparable free drugs [21]. Most important here is the size, which allows passive accumulation in the tumor, likely through enhanced permeability and retention (EPR), an observed phenomena whereby damaged or “leaky” blood vessels allow for extravasation of NPs [22]. Here, we investigate cellular/tumoral uptake of RTX-laden nanoparticles (NPs) in a murine CRC model when administered intravenously to investigate the impact of RTX or raltitrexed with FA as a targeting ligand. Improved understanding of the impact of functionalizing NP surfaces on their eventual intracellular trafficking in vivo may lead to better treatment outcomes clinically, and will likely rely on investigation of specific receptor-ligand mechanisms on an individual cell type basis [23]. Early steps in determining this lie in developing systems that can be used to track NP fate, either through fluorescence, chemical composition, or other means. The presence of a near-infrared (NIR) dye loaded entrapped in a functionalized NP allows for detection of NPs by preclinical imaging systems, both in vivo and ex vivo [24,25,26]. By investigating the differences in accumulation in tumors by varying the targeting ligand compositions, improved targeting can be identified for future therapies.

The NP surface can be functionalized with targeting ligands [22,27]; here, FA or an anti-folate drug (or both) can be adsorbed to the surface of the particle. By adsorbing various polyelectrolytes to the surface of the NP, encapsulation and targeting can be achieved [28]. This layer-by-layer (LbL) assembly strategy has been used for encapsulating fluorophores or imaging agents, therapeutics, and/or targeting ligands. Once a layer is applied, additional layers can be added sequentially after purification of excess polyelectrolyte, through centrifugation or tangential flow filtration (TFF) [29]. To develop a theranostic NP with a NIR fluorescent core and functionalized surface, we applied LbL technology to the coating of commercially available NIR dye-containing polystyrene beads. Once functionalized with an outer layer RTX or RTX/FA, the changed uptake and viability are examined in vitro, and the biodistribution versus uncoated (bare) particles is examined in vivo. The ease of changing the terminal layer allowed us to examine the differences between RTX alone and in combination with FA to investigate differences attributed to the terminal layer composition.

## 2. Materials and Methods 

### 2.1. Materials

Raltitrexed, and poly-l-lysine hydrochloride (PLL, 15–30 kDa) were purchased from Sigma Aldrich. Folic acid was purchased from Fisher Scientific. All LbL methods were carried out in Milli-Q water from a Milli-Q source. KR2i TFF system was purchased from Spectrum Laboratories (Repligen, Waltham, MA, USA).

### 2.2. Layer-by-Layer Process

Nanoparticle suspensions containing 2 wt% solids were sonicated (3 applications of 20% amplitude for 10 s on a Branson 450 digital sonifer (Branson Ultrasonics, Danbury, CT, USA)) and diluted to 0.2 mg/mL, and 3 mL was aliquoted into 15 mL conical tubes. The 3 mL sample was mixed briefly with 3 mL of 1 mg/mL PLL solution, and again sonicated 3 times at 20% amplitude for 10 s. The feed and retentate lines from the TFF setup were then placed into the conical tube, with the feed line near the bottom of the conical tube. Milli-Q water was then added to nearly fill the conical tube. The TFF system was then activated, beginning the fluid circulation through the system. As the excess PLL exited as permeate, the liquid level in the conical tube descended. Once the meniscus approached <3 mL, the tube was again filled with Milli-Q water to further wash the circulating nanoparticles. The liquid level was allowed to reach the bottom of the tube, where the flow in the system was paused, the external portion of the feed line was rinsed with water, and immersed in the water. The pump was activated, and the liquid level was returned to the original volume. The system was then flushed with water. The PLL-coated particles were added to a mixture containing either RTX or RTX and FA. Mixtures were as follows: RTX—3 mL of 0.5 mg/mL with 1 mL of 0.5 M NaOH, RTX/FA—2.5 mL of 0.5 mg/mL RTX, 2.5 mL of 0.5 mg/mL FA with 1 mL of 0.5 M NaOH. Once brief mixing occurred, the mixture was purified using the same strategy as described for PLL.

### 2.3. Nanoparticle Characterization

Size and zeta potential measurement were acquired using a Malvern Zetasizer ZS (Malvern Panalytical, Malvern, UK). Measurements were taken in triplicate in DTS 1070 zeta cells (Malvern), with values presented as the mean ± standard deviation of the measurements. Transmission electron microscopy (TEM) was used to further observe size and morphology of nanoparticle formulations. Images were acquired sing a Technai F-20 TEM operating at 4200 eV. Grids (Ted Pella, Redding, CA, USA) with copper substrate-formavar/carbon backed were used to deposit 10 µL of NP solution dropwise. Following dropwise addition of NP solution to the grid, the grids were placed in a desiccator overnight to allow for the surface to dry. Infrared spectroscopy was carried out using a Nicolet iS5 spectrophotometer (ThermoFisher, Waltham, MA, USA). Freshly prepared NP solutions were lyophilized, and the dry powder was compressed into a diamond filament. Total internal reflectance measurements were used to acquire the spectrum for each formulation at 32× resolution between 4000 and 400 cm^−1^. 

Drug loading and encapsulation efficiency were determined via high performance liquid chromatography (HPLC). To determine drug content, nanoparticle solutions containing RTX and RTX/FA NPs with excess polyelectrolytes were passed through a 100 kDa spin filtration unit (Pall). The samples were spun at 5500 rpm for 5 min, which allowed the drug to exit as filtrate while retaining the NPs in the retentate. Filtrate samples were run on a Shimadzu SPD-20A HPLC instrument (Shimadzu, Torrance, CA, USA) equipped with a UV-Vis detector and an Agilent Zorbax Rapid Resolution SBC-18 column (4.6 × 100 mm, 3.5 µm, Santa Clara, CA, USA). Drawn aliquots were run without dilution. A five-minute gradient method was utilized with a flow rate of 1 mL/min and a 311 nm detection wavelength. The mobile phase increased from 65:35 methanol/water to 70:30 methanol/water over the first 2 min and held there until the method was complete with elution occurring at ~2.2–2.4 min. Loaded drug was calculated from the difference between inlet mass of drug and filtrate mass of drug.

### 2.4. Cell Uptake 

CT26 murine colorectal carcinoma cells (ATCC, Manassas, VA, USA) were cultured in RPMI 1640 (Corning, NY, USA) media supplemented with 10% fetal bovine serum and 1% penicillin/streptomycin. When 90% confluent, the media was aspirated, the cells were washed with PBS, and 0.25% trypsin-EDTA was used to detach the cells from the culture flask. The cells were diluted in media once and counted using a Countess II FL Automated Cell Counter (ThermoFisher). The cells were then prepared for either cell uptake or cell viability assays.

To evaluate enhanced uptake of the coated NPs versus bare particles, uptake in CT26 cells was evaluated by flow cytometry and fluorescence microscopy. Utilizing the culture conditions described above, 10^6^ cells were plated into each well of a 6-well plate, followed by 2 mL of RPMI media. The cells were allowed to settle overnight. The following day, the media was aspirated, NP solutions (using with fluorescent core of 660/680 dye) were diluted in media and added to each well. A control well was left untreated. The cells were placed in an incubator for 4 h to allow uptake of the NPs. Following uptake, the cells were prepared for either flow cytometry or fluorescence microscopy. 

To prepare the cells for quantitative assessment of cell uptake using flow cytometry, the cells were washed with PBS, detached using trypsin, and diluted in media. The cells were centrifuged at 500× *g* for 5 min to form a pellet. The media was aspirated, followed by redispersion of the cells in 1 mL of PBS. The cells were pipetted into polystyrene tubes through cell strainer caps to remove cell aggregates. Using a MACSQuant flow cytometer (Miltenyi Biotec, Cologne, Germany), cellular fluorescence of no treatment control, bare CML NP-treated, and coated NP treated cells was determined sequentially. The NP fluorescence was determined using a 632 nm excitation laser line, with a 670/30 nm emission/bandwidth filter. Forward scattering and side scattering were used to determine single cells passing through the detector, and 10^5^ events (or cells) were passed through the detector to establish a distribution for each sample in each treatment group. 

To prepare the cells for fluorescent imaging, the cells were washed with PBS twice, and 2 mL of 10% formalin solution was added to each well, and allowed to fix for 30 min. Once fixed, the cells were washed twice with PBS, followed by addition of PBS containing DAPI and AlexaFluor 488-phalloidin dyes (ThermoFisher), and allowed to stain for 30 min. After staining, the cells were washed once, and the wells were filled with 2 mL of PBS. Images could then be acquired using an EVOS FL AUTO II (ThermoFisher), with filter cube sets able to capture the nucleus (blue—DAPI), cell body myelin filaments (green—AlexaFluor 488), and the NPs (red—660/680 dye).

### 2.5. Cell Viability

CT26 cells were cultured as described in the previous section. The cells were further diluted to 20,000 cells/mL, then plated at 2000 cells per well in a 96-well plate and allowed to attach overnight. After seeding, media containing NP solution was added to the majority of wells, with half-fold serial dilutions across the plate, in triplicate. The final wells were dosed with DMSO and no treatment (i.e., media), in triplicate, for the positive and negative controls. After 72 h, 10 µL of Alamar Blue cell viability reagent (ThermoFisher) was added to each well. After 1.5 h, the fluorescent signal was determined at 560/590 excitation/emission wavelength using a Tecan M200 Infinite plate reader (Tecan Trading AG, Männedorf, Switzerland).

### 2.6. In Vivo Biodistribution

Twenty 6-week-old BALB/c mice (Charles River Laboratories, Wilmington, MA, USA) housed in modified barrier animal facilities prior to tumor inoculation. On the day of tumor inoculation, cell suspensions containing 6 × 10^6^ CT26 cells/mL were prepared in sterile PBS for injection. Fifty-microliter injections were made into the right hind flank of isoflurane-anesthetized mice, implanting a total of 3 × 10^5^ cells subcutaneously. The tumors were allowed to grow for 2–3 weeks to reach a threshold size of around 100 mm^3^ (measured twice weekly in this 2–3 week period by calipers, using formula 0.5 × long length × short length^2^). 

After the tumors in the majority of animals had reached the desired size, animals were randomized into four groups, with average size roughly even among the groups. The saline group contained two animals, the bare group contained three animals, and the RTX and RTX/FA nanoparticle groups contained four animals. Two-hundred-microliter injections of either saline, bare NPs, RTX NPs, or RTX NPs (using 715/755 fluorescent dye cores) were injected via tail-vein once per day for 3 days, roughly 24 h apart. Twenty-four hours following the third injection, animals were sacrificed, organs (including tumors) were extracted, and immediately imaged using an IVIS XRMS III imaging system (PerkinElmer, Waltham, MA, USA). Sequences of images of arranged organs were imaged at 1 s, 3 s, and 6 s exposure time with imaging settings of medium binning, f1, and filter positions at 720/790 for excitation and emission. Following initial image acquisition, all organs were immersed and stored at 4 °C in 10% formalin in PBS for 48 h, followed by immersion and storage in 70% ethanol. After two days in the ethanol, all tumors were arranged by treatment group and imaged in the IVIS again, with a higher exposure of 20 s in order to determine relative fluorescence away from the other organs. All animal experiments followed guidelines set forth by Oregon Health and Science University (OHSU) Institutional Animal Care and Use Committee.

Following imaging of the ex vivo tumors, the samples were placed in histology grids and labeled, and transferred to the Histopathology Shared Resource at OHSU. Samples were paraffin embedded, sliced, and prepared according to the required staining protocol, which included either no staining, hematoxylin and eosin (H & E), or caspase-3 (CC3, Promega, 1:1500 dilution). Slides produced from staining were transferred to the Advanced Light Microscopy Core at OHSU. Scanned images of each slide were taken on a Leica Axios Imaging system (Leica) using a 10× objective. All tumors were stained and imaged; complete sets of organs were stained and imaged from one representative animal per group. Unstained slices were scanned using the 700 nm setting on an infrared imaging scanner (LI-COR, Odyssey, Lincoln, NE, USA) with low pixel resolution.

### 2.7. Statistical Analysis

Data are presented as mean ± standard deviation. Statistical significance was evaluated with student *t*-test using GraphPad Prism 8 software. *p* < 0.05 was considered statistically significant, with further confidence indicated by asterisks (*, *p* < 0.05, **, *p* < 0.01, ***, *p* < 0.001, ****, *p* < 0.0001).

## 3. Results and Discussion

### 3.1. Drug Encapsulation Process

**Figure 1:**
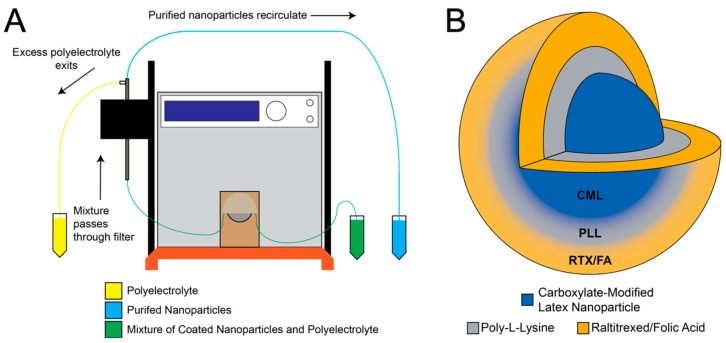
Tangential flow filtration (TFF) allows for rapid purification after layering of various materials onto the surface of carboxylate modified polystyrene latex (CML) nanoparticles (NPs). (**A**) Schematic representation of TFF system used to purify CML from poly-l-lysine (PLL), followed by separation of the NPs from raltitrexed (RTX) or RTX/folic acid (FA) mixture. (**B**) Illustration of the Layer-by-layer (LbL)-coated NP formulation.

Layer-by-layer deposition onto NP substrates using alternating charged polyelectrolytes has been employed previously for numerous applications [29,30,31]. Here, we use the process to attach FA and anti-folate drugs to the surface of polystyrene substrates. This process is accelerated through the use of a TFF setup, as described in the methods section. Each layer can be added within a few minutes. A schematic of the layering process is shown in Figure 1A. The completed NP contains a fluorescent dye (either 660/680 or 715/755) terminated with a therapeutic and targeting ligand (RTX or RTX/FA), as depicted in Figure 1B.

### 3.2. Nanoparticle Characterization

**Figure 2:**
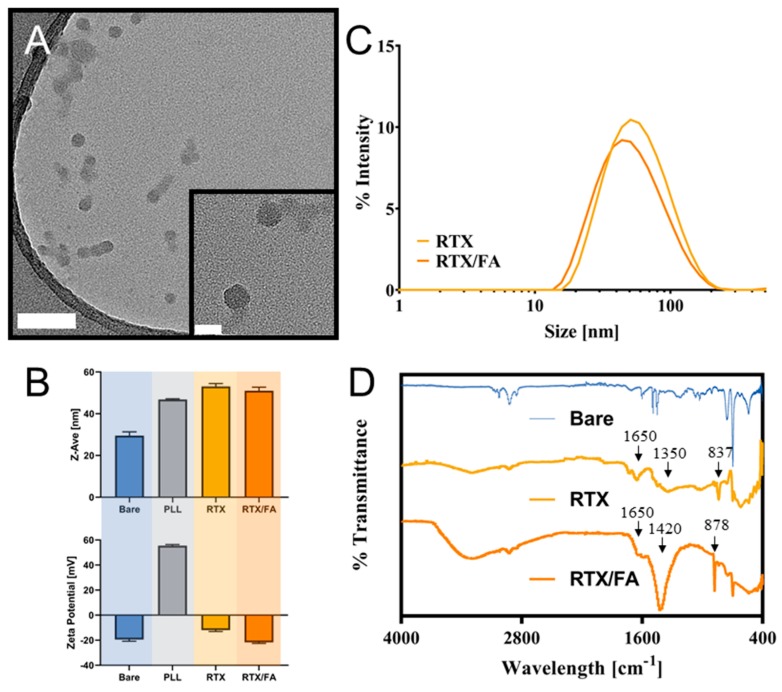
Characterization of RTX and RTX/FA NP formulations. (**A**) TEM images of formed RTX/FA NPs. Scale bar in lower magnification (larger image)—100 nm, scale bar in higher magnification image (inset)—20 nm. (**B**) Intensity derived distribution for the formed NPs as measured by dynamic light scattering (DLS). (**C**) Mean size and zeta-potential measurements at each step in the layering process shows increase in size during synthesis, and accompanying alternating charge. (**D**) IR spectra for the formulated NPs.

The size and morphology of the NPs was evaluated by TEM. Images of the RTX/FA coated NPs taken using TEM are shown in Figure 2A (RTX NPs are shown in Supplementary Info, Figure S1). Dynamic light scattering (DLS) measurements yielded size distributions for the formed particles, as shown in Figure 2B. The highest populations were approximately 40–50 nm in size, with the RTX/FA NPs appearing slightly smaller in overall population distribution. Z-Ave measurements from DLS measurements are shown in Figure 2C. The particle cores appear to be roughly 20–30 nm in diameter, which is consistent with DLS measurements (Figure 2C). The size and zeta potential of each layer addition was monitored during NP synthesis (after each purification step) to confirm that the layers were being formed as intended, also shown in Figure 2C. For the RTX NPs, the size does not increase largely across the synthesis, starting at intensity weighted mean hydrodynamic diameter, or Z-Ave, of 29.53 ± 1.81 nm, and finishing at a Z-Ave of 53.08 ± 0.33 nm (PDI = 0.265 ± 0.04). For the RTX/FA NPs, the Z-Ave of the final formulation is 51.02 ± 1.70 nm (PDI = 0.215 ± 0.004). The zeta potential, though, displays marked shifts from negative (CML surface) to positive (PLL coating) to negative (drug and/or folate terminal layer). This flipping of the zeta potential has been seen in similar systems [28,30,32], and is a simple way to ensure the layer is deposited on the surface, especially for smaller polymer deposition where the size may not change significantly. IR spectroscopy was used to identify changes in characteristic wavelengths due to the presence of the molecules added to the surface of the NPs. The waveforms for the synthesized NPs are shown in Figure 2D. The two formulations seem to add different peaks to the measured waveforms, with the most distinct changes appearing at ~800–900 cm^−1^, 1300–1500 cm^−1^, and 1600–1700 cm^−1^. The 800–900 nm peaks could be additional contributions to alkene bonding, as are most of the early peaks. The other two distinct peaks are likely the addition of the oxygen functional groups, with carboxylic acids added through the glutamic section of both RTX and FA likely contributing strong in these regions. Nitrogenous functional groups are likely to contribute in these regions as well.

Raltitrexed concentrations were estimated as 148 µg/mL in the RTX formulation and 129 µg/mL in the RTX/FA formulation (with a concentration of 118 µg/mL FA present in the RTX/FA formulation). This yields nearly identical encapsulation efficiencies of 29.7% for the RTX NPs and 30.9% for the RTX/FA NPs (calculated as the mass of RTX present on the final particle/mass of RTX present when coating the PLL layer). The release of the RTX from the NPs is anticipated to be rapid, as this was observed with a similar particle formulated previously [31], with >80% of the therapeutic released in the first 8 h. 

### 3.3. Cell Uptake

**Figure 3:**
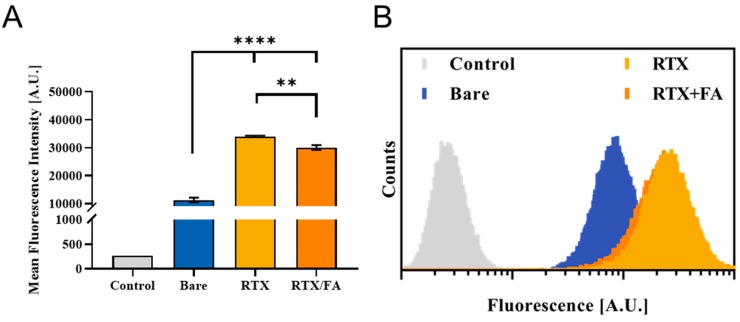
Cellular uptake of RTX and RTX/FA NPs in vitro via flow cytometry. (**A**) Mean fluorescence intensities of cell populations analyzed using flow cytometry show that the addition of the therapeutic alone and in combination with FA lead to higher uptake. **, *p* < 0.01, ****, *p* < 0.0001. (**B**) Representative histograms from each treatment group show marked shift in populations with the addition of the targeting layers. (RTX and RTX/FA are very similar in overall distribution, with most of the RTX distribution obscuring the view of the RTX/FA distribution.).

To investigate the effect of the terminal layer presence on the CML substrate for cell-mediated uptake purposes, two sets of experiments were run. In the first, a quantitative assessment of NP fluorescence was acquired using flow cytometry. CT26 murine CRC cells were dosed with 5 µg/mL (CML) of particles for 4 h, washed with PBS, and fixed in 10% formalin. After fixation, the cells were washed again, and kept at 4 °C until their fluorescence could be determined using flow cytometry. A MACSQuant (Miltenyi Biotec) cytometer was used to determine NP fluorescence of ~100,000 cells, gating for single cells. In Figure 3A, the mean fluorescence intensities from the cell populations of the various groups are shown. The bare NPs show a markedly higher signal above background (from no treatment control), while both types of layered particles showed a 2–4-fold higher cell uptake over the 4 h exposure period, indicating that the addition of the terminal layer for both particles led to a statistically significant increase in cell uptake. RTX/FA coating had a lower signal on average than the RTX coating alone. This difference was found to be significant. The addition of the RTX and FA to the terminal layer was intended to further increase the uptake both in vitro and in vivo, but inverse effect was observed in vitro. RTX could have a higher uptake potential in CT26 cells, or the combination with FA may interfere with specific uptake mechanisms that reduce the overall uptake. In general, the fate of the individual NPs once they enter the media environment en route to the cells is not completely understood. Factors such as size due to aggregation, apparent surface charge, and location of RTX and FA on the surface of the particles could all influence the intracellular trafficking of the NPs, both in vitro and in vivo [23]. Representative individual histograms for the flow cytometry data are shown in Figure 3B. 

**Figure 4:**
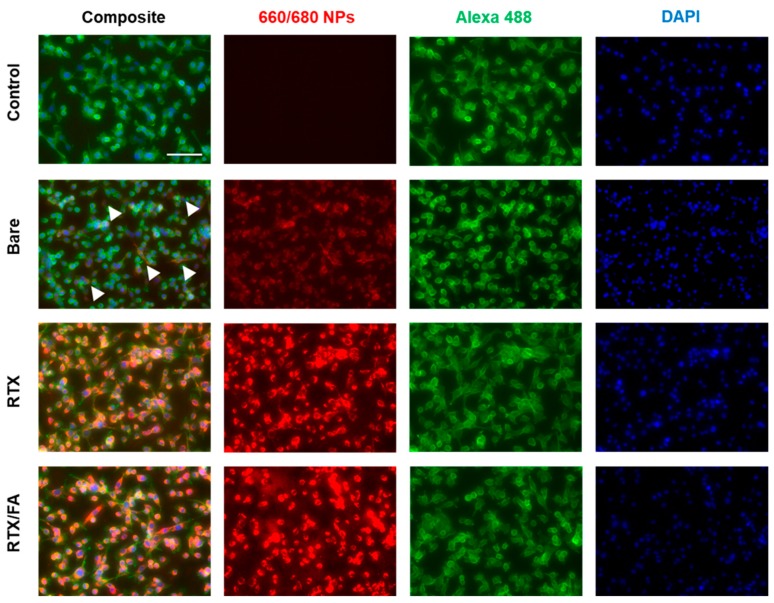
Cellular uptake of LbL NP formulations in vitro via fluorescence microscopy show an increase in red signal due to higher uptake of NPs, which is highest in the RTX and RTX/FA images. Scale bar indicates 100 µm.

The populations shift from left to right with increasing fluorescent signal from the red fluorophore in the NPs, with the RTX and RTX/FA NPs showing highest signal intensities (with significant overlap in their populations, leading to the RTX/FA distribution being covered by the RTX distribution in the figure). To visualize the cell uptake of the NPs qualitatively, the second assessment used fluorescence microscopy. CT26 cells were plated, dosed for 4 h, and fixed to the surface of a six well plate rather than in suspension. The cells were stained with DAPI and Alexa 488-phalloidin (blue and green respectively) prior to imaging on an EVOS FL Auto II (Invitrogen, Waltham, MA, USA). Images taken from wells treated with each NP are shown in Figure 4. 

A no treatment well shows no red fluorescence, while bare CML particles show low signal, indicated by arrows in the image. The wells treated with the coated particles show much brighter signal colocalized within the cells, indicative of uptake quantified by flow cytometry. Unlike flow cytometry, though, the difference in signal intensity between the different coating groups is indiscernible.

### 3.4. Cell Viability

**Figure 5:**
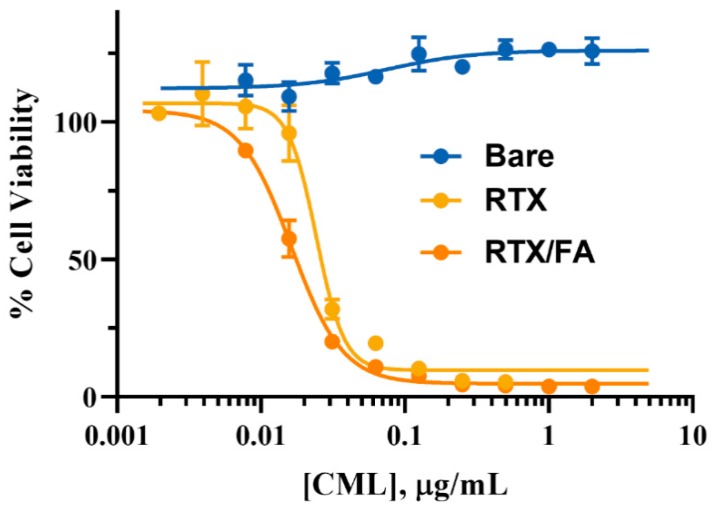
Cell viability of CT26 cells after 72 h exposure to various NPs. Toxicity is increased greatly in the formulations that contain RTX, with no observed toxicity in cells treated with the uncoated NPs.

The efficacy of drug-coated formulations was evaluated in vitro using a cell viability assay, with Alamar Blue (Invitrogen) as the reagent. CT26 cells were dosed with NPs diluted in media of the various formulations for 72 h, after which the reagent was added to the wells. After 90 min incubation with the reagent, the fluorescence from the wells was read using a plate reader. Wells containing viable cells have a proportionally higher fluorescent signal, and are presented as a proportion of a negative control (untreated cells). Cells unaffected by the treatment show close to 100% viability, while lower viability can be attributed to toxic effects of the NPs. The results of this assay are shown in Figure 5. Bare CML NPs do not contain a therapeutic, and thus show higher viability even at the highest concentrations of dosed NPs. The RTX and RTX/FA NPs show greater toxicity, with an IC_50_ values of 0.024 and 0.016 µg/mL, respectively (free RTX was found to have an IC_50_ of 14.44 nM at 72 h). These values are roughly the same, and the curves appear similar overall, indicating that their lethality may be similar in vivo as well, even though their terminal layer is different. The disparity between the free drug IC_50_ and the NP IC_50_ (the free drug IC_50_ value is roughly a factor of 10 less than the NP version) could be due to release kinetics or reduced efficacy due to the adsorption to the NP substrate. This is only an in vitro assessment, and the NP may have higher potential to be efficacious in vivo, where the NP will aid in accumulation at the target site.

### 3.5. Biodistribution 

**Figure 6:**
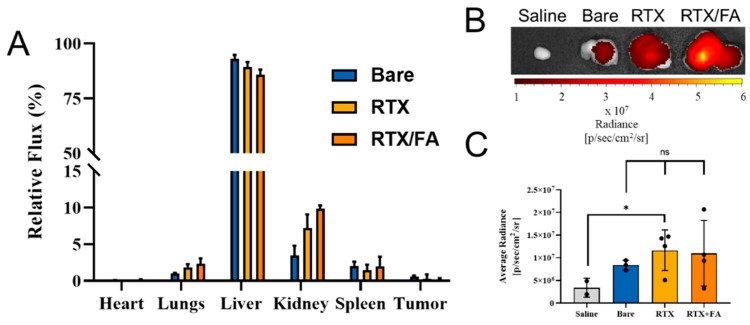
Biodistribution of NP formulations injected into mice bearing CT26 tumors. (**A**) Mean relative flux in each organ across all animals in each treatment group. Liver showed high accumulation, with small percentages in the lungs, kidneys, and spleen. (**B**) Representative tumors from each treatment group show increased signal when imaged at a higher exposure time. (**C**) Mean average radiance of all tumors imaged at higher exposure time, with bare, RTX, and RTX/FA-treated organs showing higher average signal due to the NPs. *, *p* < 0.05.

Mice bearing CT26 tumors were dosed with 3 × 200 µL injections of saline, bare CML NPs, RTX NPs, or RTX/FA NPs. One injection was made daily over the course of 3 days. After the last injection (24 h), the animals were sacrificed, the organs and tumors were removed, and imaged on an IVIS XRMS III system (PerkinElmer). Total radiant flux from each set of organs was acquired using the associated software (Living Image), and was used to determine the relative percentage of recovered fluorescence from the organs. These values are shown in Figure 6A. 

The majority of the NPs for each treatment group accumulates in the liver, with small amounts present in the lungs, spleen, and kidneys. The heart and tumors show significantly lower amounts of the injected NPs. While it is common to see small percentages of the injected material in the tumor, one of the main aims of this work is to further investigate the potential difference in uptake in the tumors. To further investigate this difference, the tumors were isolated from the other organs, and imaged together with a longer exposure. Representative tumors from each treatment group are shown in Figure 6B. The signal increases by at least one order over the saline group. The difference in radiance from the NP treatment groups is difficult to interpret from the image, and thus average radiance of the tumor tissue was examined in order to quantify the relative number of NPs in each treatment organ (Figure 6C). While not significant, there appears to be a small difference in the mean values across the groups, with the RTX and RTX/FA NPs showing slightly higher average values, likely due to the presence of the terminal targeting layers. Furthermore, the RTX group appears to show the majority of the tumors as having a higher average radiance than the bare NPs, with the mean value brought down by a single tumor that shows little fluorescence. Overall, these are low sample sizes, and significance cannot be drawn from these results, but they may indicate that this work could be repeated with larger groups to fully elucidate the usefulness of these targeting ligands on the NPs. 

**Figure 7:**
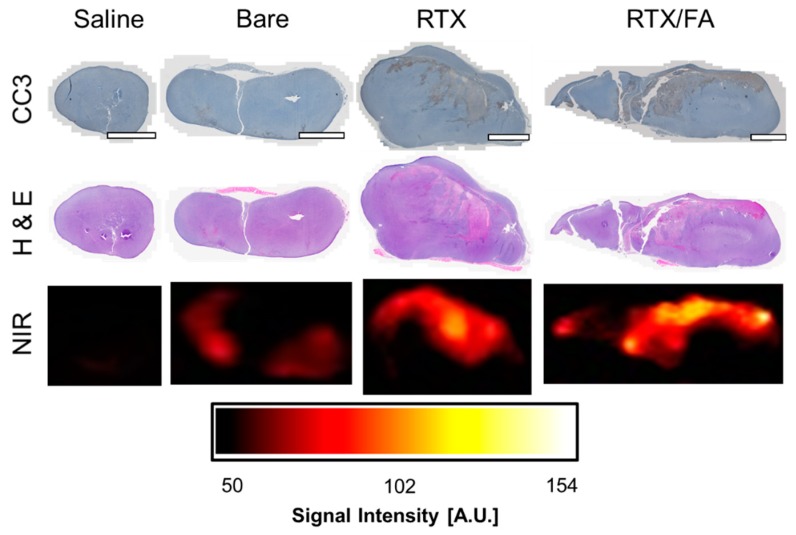
Tumor toxicity associated with NP treatments. Cleave-caspase 3 and H & E stained representative tumors show potential in vivo toxicity of the RTX and RTX/FA formulations. Scale bar indicates 1 mm. Brown color indicates regions of apoptosis, which are more prevalent in the RTX and RTX/FA-treated organs. Unstained slides were scanned at lower resolution using a LI-COR Odyssey fluorescent scanner at the 700 nm setting, which appear to show higher signal in regions that showed higher cell death in similar slices.

Organs and tumors from all treatment groups were prepared for histology, paraffin embedded, sliced, and stained with H & E and CC3. Tumors stained with CC3, shown in Figure 7, showed some toxicity from the RTX and RTX/FA treatment groups, with certain area of the tumor showing brown staining indicative of apoptosis. Adjacent slices stained with H & E potentially show the same trend, with reduced dark purple color indicating reduced nuclear staining. Accumulation of NPs could be inferred from higher fluorescent signal in accompanying areas (from adjacent unstained slides), measured using a LI-COR Odyssey fluorescent scanner. Here the heterogeneous uptake of NPs can be seen in all treated tumor samples. Although only qualitative due to small sample size, the general trend of overall tumor accumulation represented by these cross-sections is consistent with whole tumor ex vivo imaging. Importantly, co-localization of the fluorescence signal with regions of apoptosis imply NP-mediated delivery of the RTX. This is likely not the only contributor to the apoptotic regions, though, as the overall larger size of the tumors indicates that there could be apoptotic regions due to deoxygenation from rapid growth of the tumor. The sample sizes were small for this work, with inconsistent tumor volumes across the groups. In future work, the sample sizes would be larger with similar tumor sizes across all of the groups, most likely starting at a smaller tumor size to eliminate potential tumor core apoptosis due to lack of oxygen/nutrients.

**Figure 8:**
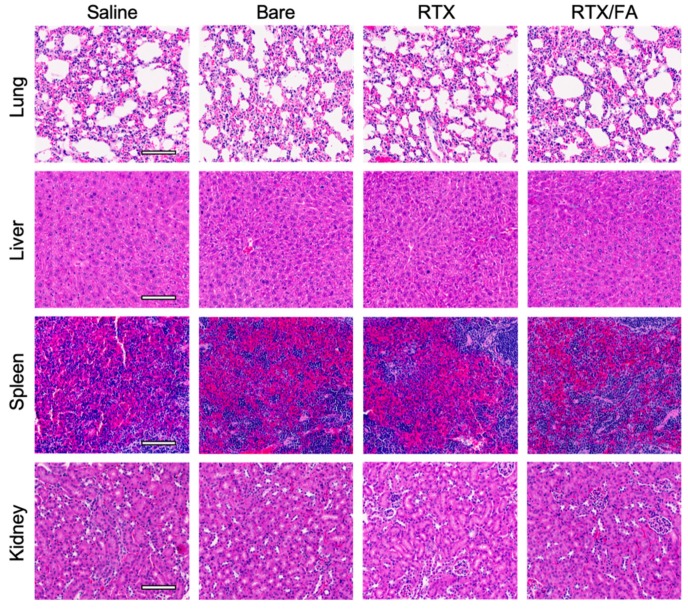
Clearance organ toxicity associated with NP treatments. Images of organs stained with H & E show little to no difference between saline treated animals versus NP treated animals. Scale bar indicates 100 µm.

To investigate systemic toxicity due to NP treatment, clearance organs from a representative animal from each treatment group were stained with H & E. Images from each organ are shown in Figure 8. The accumulation in these organs, indicated from the IVIS imaging, does not seem to convey noticeable systemic toxicity. Spleen uptake of the NPs was observed (Figure 6A), with little to no difference in the red or white pulp regions of the tissue samples. In the kidneys, another region that displayed fluorescence from the NPs, the tissue is near identical to the saline control samples. Raltitrexed was developed in part to reduce renal toxicity of previous generations of this class of drugs [9,10]. This, in combination with its attachment to a nanocarrier, may help further reduce off-target toxicity of the therapeutic.

## 4. Conclusions

The NPs described in this work highlight the ease of LbL assembly for attachment of therapeutics and targeting ligands. By varying the terminal layer of the NPs, we assessed the cell uptake and biodistribution of coated NPs versus uncoated controls. Based on flow cytometry and fluorescence microscopy, we observed enhanced uptake due to the RTX and RTX/FA terminal layers. The NPs were also functional, in that they showed cytotoxic effects on CT26 CRC cells. When administered intravenously, the different formulations accumulated in various organs and the implanted tumors ex vivo. The animals treated with RTX and RTX/FA NPs showed slightly higher tumor accumulation on average than uncoated NPs. Histology showed no observed off-target toxicity of the formulations, with possible higher accumulation of the fluorescent NPs in the unstained slides of the RTX and RTX/FA treatment groups. With higher samples sizes and higher dosing of the NPs, these formulations may be optimized for improved uptake in tumors over-expressing α-folate receptors.

## Supplementary Materials

The following are available online at https://www.mdpi.com/1999-4923/12/2/133/s1, Figure S1: Transmission electron microscopy images of formed RTX NPs. Scale bar in lower magnification (larger image)—50 nm, scale bar in higher magnification image (inset)—20 nm.
