# Supplementary material for: Formulation of Folate-Modified Raltitrexed-Loaded Nanoparticles for Colorectal Cancer Theranostics
Source: Pharmaceutics. 2020 Feb 5;12(2):133. doi: 10.3390/pharmaceutics12020133 (PMC7076500; doi:10.3390/pharmaceutics12020133)
Supplement: Supplementary file 1 [file pharmaceutics-12-00133-s001.pdf]

pharmaceutics

Supplementary Materials: Formulation of Folate-Modified Raltitrexed-Loaded Nanoparticles for Colorectal Cancer Theranostics

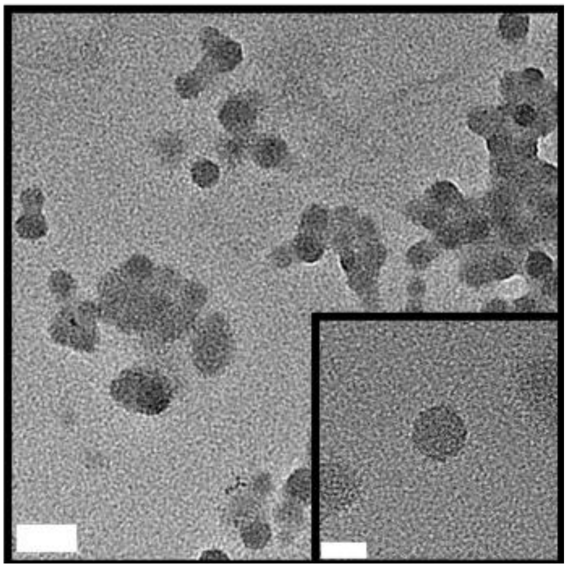

Figure S1. Transmission electron microscopy images of formed RTX NPs. Scale bar in lower magnification (larger image)—50 nm, scale bar in higher magnification image (inset)—20 nm.
